# Fatigue is distinct from sleepiness and negatively impacts individuals living with obstructive sleep apnea (OSA): results from qualitative research of individuals with OSA

**DOI:** 10.1186/s12955-025-02355-1

**Published:** 2025-03-24

**Authors:** Helene A. Emsellem, Hilary H. Colwell, John Cronin, Ronald H. Farkas, Susan D. Mathias

**Affiliations:** 1 Sleep Health Institute, 10221 River Road, PO Box 61324, Potomac, MD 20859 USA; 2 Health Outcomes Solutions, 1149 Crystal Drive, Palm Beach Gardens, FL 33418 USA; 3 Apnimed, Inc., 39 John F. Kennedy St., 4th Floor, Cambridge, MA 02138 USA

**Keywords:** Obstructive sleep apnea, Excessive daytime sleepiness, Fatigue, Patient reported outcomes, PROMIS, Qualitative research, Cognitive debrief, Concept elicitation

## Abstract

**Background:**

We sought to identify important issues regarding symptoms and impacts of obstructive sleep apnea (OSA), to explore fatigue and sleepiness, and evaluate the content, clarity, and relevance of specific patient reported outcome (PRO) measures.

**Methods:**

Participants in the US with OSA and at least mild fatigue were studied. Individuals with positive airway pressure (PAP) therapy intolerance or current PAP refusal (non-PAP users) and those who initiated PAP within the past 12 months (PAP users) were identified and interviewed. Interviews included concept elicitation questions about symptoms and impacts of OSA. Participants then completed several PRO measures (the PRO Measurement Information System [PROMIS] Fatigue-8a, PROMIS Sleep-Related Impairment-8a, Epworth Sleepiness Scale [ESS], Patient-Global Impression of Severity of Fatigue [PGI-S Fatigue], and Patient Global Impression of Change in Fatigue [PGI-C Fatigue]) and were cognitively debriefed to evaluate their content, clarity, and relevance.

**Results:**

A total of 30 individuals with OSA (20 non-PAP and 10 PAP) were enrolled. In addition to fatigue (reported by 100%), sleepiness (75%), difficulty concentrating (85%), dry mouth/throat (60%), headaches (50%) and interrupted sleep (50%) were the most common symptoms reported by non-PAP users. Fifty-eight percent of non-PAP users rated fatigue as the most bothersome symptom; 5% rated sleepiness as the most bothersome. Among PAP users, the most common symptoms (prior to PAP use) in addition to fatigue (100%) were sleepiness (90%), difficulty concentrating (60%), dry mouth/throat (60%), headaches (50%), and interrupted sleep (50%). Fatigue was rated as most bothersome by 56% of PAP users; sleepiness was rated as the most bothersome by 22%. All participants mentioned fatigue and sleepiness separately, indicating they are considered distinct symptoms. In general, participants found the PRO measures to be relevant and clear, and results supported their content validity, clarity, and relevance.

**Conclusions:**

Fatigue was the most bothersome symptom reported by non-PAP and PAP users. Participants described fatigue as a distinct and different concept from excessive daytime sleepiness. Participants reported that their OSA symptoms negatively impact daily activities, functioning, work, and relationships. The PRO measures are clear and relevant for individuals with OSA and appropriate for use in both clinical and research settings.

## Introduction

Obstructive sleep apnea (OSA) is the most common sleep-related breathing disease, affecting more than 50 million people in the US and nearly one billion individuals worldwide [1–3]. OSA is characterized by sleep-related neuromuscular dysfunction and predisposing anatomic abnormalities, leading to recurrent partial or complete upper airway collapse during sleep that results in decreased oxygenation [4–6]. The negative sequalae of OSA is significant, resulting in increased likelihood of cardiovascular disease, hypertension, stroke, diabetes, depression, motor vehicle accidents, as well as early mortality [7–9]. 

Common symptoms of OSA include excessive daytime sleepiness, loud snoring, and gasping or choking at night [10]. One of the most commonly cited symptoms is excessive daytime sleepiness [11, 12]. Excessive sleepiness has been linked to higher comorbidity and greater impairment in productivity [13]. Fatigue or decreased energy have been reported as common and important symptoms in OSA; one study in individuals with OSA reported fatigue or decreased energy in 71% and 80%, respectively [14]. Studies of fatigue have shown that it is associated with poorer respiratory-specific health-related quality of life (HRQoL) [14, 15]. Additionally, individuals with OSA have indicated that fatigue is a more important symptom than sleepiness [14, 15], and there is a growing body of literature noting the importance of distinguishing fatigue from sleepiness in this population [16–21]. 

Multiple studies have examined the relationship between sleepiness and fatigue in populations of OSA and sleep disorders and concluded that they represent distinct concepts [20, 22]. Suh, et al., in particular, reported only moderate correlations between measures of sleepiness and fatigue in 60 patients with sleep disorders [20]. The need for more precise definitions of sleepiness and fatigue has been noted, suggesting that sleepiness reflects “drowsiness, sleep propensity, and decreased alertness.” [19] Fatigue, on the other hand, can include “weariness, weakness, and depleted energy,” [19], or can be described as “tiredness and lack of energy without increased sleep propensity.” [23].

There are a variety of available treatments for OSA [10], including the current frontline standard of care, positive airway pressure (PAP) therapy, which temporarily opens the airways but does not address the underlying neuromuscular dysfunction in OSA. However, many patients refuse treatment, and more than 50% who are willing to try PAP are intolerant or non-adherent with PAP use for a variety of clinical and psychological reasons [24, 25]. The impact of PAP on reducing fatigue and sleepiness is mixed [26, 27] and appears to be affected by patients’ adherence and number of hours per night of PAP use [28]. The lack of acceptability and tolerability of current OSA treatments, such as PAP therapy, indicates an unmet need for novel OSA treatment options to improve both fatigue and sleepiness.

Recently, clinical trials have shown that the combination of aroxybutynin and atomoxetine, called AD109, demonstrated significant and meaningful improvement in OSA [29, 30]. One of these trials, (MARIPOSA), was a Phase 2, randomized, double-blind, placebo-controlled, parallel arm study to evaluate AD109 in participants with mild to severe OSA [30] that also incorporated several patient-reported outcome (PRO) measures to assess patient experiences with OSA and OSA treatment. In the MARIPOSA trial, a significant improvement in Patient Reported Outcomes Measurement Information System (PROMIS) Fatigue-8a scores was observed in participants receiving AD109 compared with the placebo group. Ongoing Phase 3 studies (clinicaltrials.gov NCT05811247 and NCT05813275, respectively) include PRO measures, including the PROMIS Fatigue-8a, PROMIS Sleep-Related Impairment-8a, the Epworth Sleepiness Scale (ESS), a Patient-Global Impression of Severity of Fatigue (PGI-S Fatigue), and a Patient-Global Impression of Change in Fatigue (PGI-C Fatigue).

PROMIS is a set of PRO measures developed from research based on item banks that allow for the evaluation and calibration of individual items [31]. PROMIS Fatigue and Sleep Disturbance measures have previously demonstrated acceptable psychometric properties for assessing fatigue and sleepiness across various populations, including those with sleep apnea [32, 33]. These measures have been utilized in OSA studies of various treatments and have proven to be responsive to change [34–36]. Given the need to distinguish between fatigue and sleepiness in OSA, the PROMIS measures could provide much needed clarity to the burden of these symptoms in this population.

We sought to identify common symptoms of OSA and their impact on individuals with OSA, and to explore the relative frequency and importance of fatigue versus sleepiness. We also aimed to confirm the content, clarity, and relevance of the PROMIS Fatigue-8a, the PROMIS Sleep-Related Impairment-8a, the ESS, the PGI-S Fatigue, and PGI-C Fatigue, in adult participants with OSA (with and without use of PAP). Finally, we explored the experiences of participants who initiated PAP treatment within the past 12 months.

## Methods

### Data source and participants

Potential participants with OSA were identified at three sites in the US. Individuals identified from site databases and those who came to one of the sites to be seen for OSA were provided with information regarding the study. Those interested in participating in the study were asked to review and sign an informed consent form. Study sites then explained the study and evaluated each potential participant’s eligibility using a study-specific screening questionnaire. Human participants’ research approval for the study was provided by an independent, scientific review committee, WCG institutional review board (IRB).

Specific inclusion and exclusion criteria have been presented previously [30], but briefly: the study included adults (aged ≥18 years of age) with a confirmed diagnosis of OSA within the past 2 years and a fatigue score of at least “mild” on the PGI-S Fatigue item. Those with other sleep disorders, as well as those currently using implanted or wearable devices for the treatment of OSA, were excluded.

Two groups of participants were enrolled to reflect the OSA population. The first group of participants was required to have PAP intolerance or current PAP refusal (non-PAP users). These individuals most closely reflect those who will participate in planned late phase AD109 clinical trials. The second group of participants was required to have initiated PAP within the past 12 months and be using PAP for at least 4 h per day, 5 days a week. PAP users were asked about symptoms they experienced before starting PAP and the improvements they noticed after initiating PAP treatment. While PAP users will not be included in currently planned late phase trials of AD109, the inclusion of this group allowed for comparison between non-PAP and PAP users. All participants were remunerated upon completion of the interview for their time. Interviews were conducted between May 2023 and July 2023.

### Data collection and measures

Each participant completed a brief background questionnaire, including demographic questions. Each site also provided a copy of results from the most recent sleep study and completed a brief clinical case report form, containing information about the participant’s clinical characteristics, such as diagnosis, co-morbid conditions, and prior and current treatments for OSA.

Soliciting patient input during the development and evaluation of a PRO measure is recommended by the Food and Drug Administration (FDA) in a published document containing guidelines for PRO development [37–40]. Interviews were conducted using a video-conferencing platform (Zoom) by an experienced health service researcher. A semi-structured interview guide, developed specifically for this study, was used to facilitate each interview to collect participant input. During the concept elicitation portion of the interview, participants were asked open-ended questions about the symptoms and impacts they experienced. Sample questions from the guide were as follows: “Apart from snoring, what symptoms, if any, have you EVER experienced as a result of your OSA?” and “How, if at all, does having OSA impact your ability to do daily activities?” PAP users were also asked questions such as, “How, if at all, have your symptoms changed since using PAP?”

During the cognitive debriefing portion of the interview, participants completed the PROMIS Fatigue-8a, the PROMIS Sleep-Related Impairment-8a, the ESS, and PGI-S and PGI-C Fatigue items, and were asked questions about the content, clarity, and relevance of these measures, although due to time constraints, the ESS was only briefly discussed with participants. During the cognitive debriefing portion of the interview, participants were asked specifically about fatigue and sleepiness, such as “In your own words, what does ‘fatigue’ mean to you?” and “In your own words, what does ‘sleepy during the day’ mean?”

The PROMIS Fatigue-8a [41] is an 8-item, self-administered measure of fatigue. It has a recall period of the past 7 days and includes two sets of response options using 5-point Likert scales (“not at all” to “very much” and “never” to “always”). The PROMIS Sleep-Related Impairment-8a is an 8 item, self-administered measure of perceived functional impairments during wakefulness associated with sleep problems or impaired alertness/sleepiness/tiredness [42, 43]. It has a recall period of the past 7 days and includes response options using a 5-point Likert scale ranging from “not at all” to “very much.” Both the PROMIS Fatigue and Sleep-Related Impairment measures have previously been used in studies of OSA and sleep quality to assess the impact of treatment and/or changes over time [34, 35, 44]. The ESS [45] is an 8-item, self-administered measure assessing the likelihood of falling asleep in different situations. It does not include a recall period, and the response options are on a 4-point Likert scale ranging from “would never nod off” to “high chance of nodding off.” The PGI-S Fatigue assesses the severity of fatigue (“none”, “mild”, “moderate”, “severe”, “very severe”), while the PGI-C Fatigue assesses the change in the severity of fatigue (response options range from “much better” to “much worse”).

### Analyses

All interviews were recorded and transcribed for analysis purposes. A coding dictionary was developed and used in the analysis of the transcripts. The codebook was used to organize and categorize concepts of interest from the interviews and included descriptions and examples for each code to ensure consistency across coders. For example, if a participant mentioned a symptom such as fatigue, then a code for “fatigue” would be added. Under the code of “fatigue”, additional sub-codes would be added such as “frequency” (e.g., every day, weekly) or “severity” (e.g., mild, moderate, severe). Each transcript was coded by one coder, and then reviewed, summarized, and analyzed by a second coder who performed the analysis. Saturation tables were also developed to categorize each symptom mentioned by each participant (one saturation table for non-PAP users and one for PAP users). Saturation tables include all concepts and indicate which participant(s) mentioned each concept. The frequencies of each concept can then be tallied. Saturation is the point at which no new concepts are mentioned by subsequent participants. Achieving saturation provides a level of confidence that the majority of concepts have been identified.

## Results

### Participant population

Interviews were conducted with 30 individuals (20 non-PAP users and 10 PAP users). Among non-PAP users, equal numbers of men and women were interviewed. Eighty percent of participants had previously used PAP, and 20% had undergone a tonsillectomy/adenoidectomy. The most common co-morbid conditions were hypertension (50%), hyperlipidemia (35%), and allergic rhinitis (25%, Table 1).

Among PAP users (*n* = 10), all participants were currently using PAP, and 20% had undergone a tonsillectomy/adenoidectomy. The most common co-morbid conditions were hypertension (40%) and hyperlipidemia (40%). PAP users were asked to consider symptoms and impacts of OSA prior to starting PAP, and then asked about any improvements after initiating PAP.

### Concept elicitation results

The most common symptoms reported (Table 2) included fatigue (100% of non-PAP, 100% of PAP), feeling sleepy (75%, 90%), difficulty concentrating (85%, 60%), dry mouth/throat (60%, 60%), headaches (50%, 50%), and interrupted sleep (50%, 50%). Saturation, the point at which no new symptoms were mentioned, was achieved by the 11th interview in non-PAP users and by the 7th interview in PAP users. Fifty-eight percent of non-PAP and 56% of PAP users rated fatigue as the most bothersome symptom; sleepiness was rated the most bothersome symptom by only 5% of non-PAP users and 22% of PAP users. Participants used different terms to refer to fatigue and sleepiness. When reporting fatigue, in addition to using the word “fatigue,” participants also used “tired,” “exhausted,” “unrested,” and “worn out.” To report sleepiness, participants described “feeling sleepy” or wanting to “lay down and take a nap.” Given these differences and that participants often reported fatigue and sleepiness as separate symptoms, provided support that participants considered fatigue and sleepiness to be distinct concepts.

**Table 1:** Demographic and clinical characteristics

	All Participants (*n* = 30)	Non-PAP Users (*n* = 20)	PAP users (*n* = 10)
Time since Diagnosis (mo) (mean ± SD)	47.5 ± 63.9	127 ± 310	10.0 ± 5.9
Gender			
Male Female Non-binary	18 (60) 12 (40) 0 (0)	10 (50) 10 (50) 0 (0)	8 (80) 2 (20) 0 (0)
Age, yr (mean ± SD) (range)	53.7 ± 11.7 (32–73)	54.6 ± 11.4 (32–73)	52.0 ± 12.7 (38–72)
Education^1^			
Less than HS HS diploma Some college College degree Professional or advanced degree	0 (0) 4 (14) 6 (21) 12 (41) 7 (24)	0 3 (16) 6 (32) 7 (37) 3 (16)	0 1 (10) 0 5 (50) 4 (40)
Ethnicity			
Hispanic or Latino Non-Hispanic or Latino	2 (7) 28 (93)	2 (10) 18 (90)	0 10 (100)
Race			
White Black Asian	23 (77) 6 (20) 1(3)	14 (70) 6 (30) 0	9 (90) 0 1 (10)
Marital status			
Married Living with partner Widowed/divorced/separated Single, never married	16 (53) 5 (17) 5 (17) 4 (13)	8 (40) 4 (20) 5 (25) 3 (15)	8 (80) 1 (10) 0 1 (10)
Household Income			
< $25,000 $25,000 - $49,999 $50,000 - $74,999 $75,000 - $99,999 > $100,000 Decline to answer	0 2 (7) 3 (10) 7 (23) 10 (33) 8 (27)	0 2 (10) 2 (10) 5 (25) 5 (25) 6 (30)	0 0 1 (10) 2 (20) 5 (50) 2 (20)
Work status			
Full time for pay Part time for pay Don’t work for pay because of OSA Don’t work for pay, unrelated to OSA	22 (73) 3 (10) 0 5 (17)	14 (70) 2 (10) 0 5 (25)	8 (80) 1 (10) 0 1 (10)
Time (mos) since initial clinical diagnosis of OSA, mean ± SD	42.3 ± 59.7	58.2 ± 67.9	10.1 ± 6.4
BMI, mean ± SD	33.1 ± 4.4	32.6 ± 4.4	34.2 ± 4.2
Co-morbid conditions, n (%)			
Hypertension Diabetes/prediabetes Obesity Depression Anxiety Hyperlipidemia Allergic rhinitis Gastroesophageal reflux Arthritis/Osteoarthritis Hyperthyroidism Neuropathy Raynaud’s syndrome Back Pain Other^2^ (panic disorder gluten intolerance, scleroderma, rosacea, overactive bladder, atrial fibrillation, leukemia, binge eating, plantar fasciitis, asthma, attention deficit disorder, kidney stones, Factor 11 deficiency, breast cancer)	14 (47) 5 (17) 1 (3) 3 (10) 5 (17) 11 (37) 7 (23) 4 (13) 5 (17) 2 (7) 2 (7) 2 (7) 2 (7) 11 (37)	10 (50) 4 (20) 0 2 (10) 3 (15) 7 (35) 5 (25) 3 (15) 4 (20) 2 (10) 2 (10) 2 (10) 2 (10) 6 (30)	4 (40) 1 (10) 1 (10) 1 (10) 2 (20) 4 (40) 2 (20) 1 (10) 1 (10) 0 0 0 0 5 (50)
Have received PAP, n (%)			
Yes Currently receiving	26 (87) 10 (33)	16 (80) 0	10 (100) 10 (100)
Have received Oral Appliance, n (%)			
Yes Currently using	1 (3) 0	1 (5) 0	0 0
Have undergone a tonsillectomy/adenoidectomy, n (%)			
Yes	6 (20)	4 (20)	2 (20)
Have undergone a uvulopalatopharyngoplasty, n (%)			
Yes	1 (3)	1 (5)	0

^1^Missing for one non-PAP user

^2^Each co-morbid condition was reported one time

BMI, body mass index; PAP, positive airway pressure; SD, standard deviation

**Table 2:** Frequency of symptoms mentioned by participants

Symptom	Non-PAP Users (*N* = 20)	PAP Users (*N* = 10)
Difficulty concentrating	17 (85%)	6 (60%)
Dry mouth	12 (60%)	6 (60%)
Fatigue/tired	20 (100%)	10 (100%)
Gasp for air when sleeping/waking up	3 (15%)	1 (10%)
Headaches	10 (50%)	5 (50%)
High blood pressure	3 (15%)	2 (20%)
Interrupted sleep/wake up a lot	10 (50%)	5 (50%)
Irregular heartbeat	1 (5%)	1 (10%)
Night terrors	2 (10%)	0 (0%)
Nocturia	1 (5%)	0 (0%)
Phlegm/mucus	0 (0%)	1 (10%)
Ringing in ears	1 (5%)	0 (0%)
Runny nose	1 (5%)	0 (0%)
Sleepiness	15 (75%)	9 (90%)
Sore throat	7 (35%)	0 (0%)
Teeth grinding/jaw clenching	0 (0%)	1 (10%)

Data reported as n (%); PAP, positive airway pressure

**Table 3:** Symptom impacts

Impact	% Negatively Impacted		Representative quotes
	Non-PAP Users *N* = 20	PAP Users *N* = 10	
Daily activities	10 (50%)	5 (50%)	“I haven’t been able to really do the housework like I need to because I’m not motivated, I’m tired and I think about everything that I need to do and by the time I get home I just don’t have the energy to do it.” (A non-PAP user) “Yeah. I mean, you know, it’s one of those things that with being so exhausted feeling, you know, I maybe would neglect some day-to-day activities just because I just felt too tired to do them that day.” (A PAP user)
Physical functioning/activities	12 (60%)	5 (50%)	“Physically I feel exhausted all the time. I just feel like I don’t have the energy.” (A non-PAP user) “Like I’ll be honest with you, like my mornings just sucked and then like moving into the afternoons, again, it would get a little bit better, but then as soon as I ate lunch, I’d get wiped out again and then, yeah, the afternoons. I guess like the adjective that just keeps popping in my head is heavy. I just felt really, really heavy and just weighed down.” (A PAP user)
Emotional functioning	16 (80%)	6 (60%)	“Impatient. Irritable, to me, implies a kind of interaction with other humans and my response to them. So, you know, whether it’s verbal or response to a written word. Irritable because of poor sleep.” (A PAP user)
Social functioning	10 (50%)	5 (50%)	“If it’s really bad, I would postpone doing an activity or going out with friends.” (A non-PAP user) “I would say yeah. I mean I would feel like I’d be too tired to do anything even when I was with a lot of my friends. I wasn’t paying attention to kind of the conversation all the time, kind of, I would say like daydreaming-type situations or I would just be so exhausted that I just kind of didn’t include myself in conversation.” (A PAP user)
Relationships	15 (75%)	7 (70%)	“Because my wife is a light sleeper and it puts a lot of tension on us to be quite frank, you know.” (A non-PAP user) “I would say relationships with children because since I spend my weekends with them, they got very annoyed that I slept all weekend.” (A PAP user)
Work	9 (45%)	7 (70%)	“I’m tired and I’m foggy sometimes and it’s hard to concentrate. I can’t do complicated things in the morning as easily. They take a lot of concentration.” (A non-PAP user) “Yeah. I mean, it’s definitely, you know, you definitely notice productivity in that afternoon time, you know, kind of when I get that, basically that crash. After that I just put it, even when I kind of woke myself back up, I just couldn’t work at the same rate or a lot of times on the same task. Just trying to like, it was just hard to kind of finish the same task or stay as productive in the afternoons.” (A PAP user)

PAP, positive airway pressure

Both non-PAP users and PAP users reported negative impacts of symptoms (Table 3), including impacts on daily activities (50% both), physical (60% and 50%, respectively), social (50% both), and emotional functioning (80% and 60%), and relationships (75% and 70%). Impacts on ability to work for pay and work productivity (45% and 70%) were also common. PAP users reported improvements in their ability to do daily activities (80%) and in all areas of functioning after initiating PAP (range: 80–100%). Representative quotes can also be found in Table 3.

### Cognitive debriefing results

In general, participants found the PROs to be clear and appropriate (Table 4). Most participants were able to accurately paraphrase items and response options (range = 83–100% for PROMIS questionnaires, and 91–100% for PGI-S and PGI-C), and most found the questionnaires to be clear (range = 60–100% for PROMIS questionnaires and 74–82% for PGI-S and PGI-C). While some participants indicated that the wording of some questions or response options were “vague” (e.g., the word “bothered” in the item “How much were you bothered by your fatigue on average” in the PROMIS-Fatigue-8a), all participants were able to provide a response, suggesting that any issues with interpretation or clarity were minor. Most participants found the ESS to be clear (75% of non-PAP users and 70% of PAP users) and relevant (80% of non-PAP users and 90% of PAP users).

During debriefing of the PROMIS Fatigue and Sleep-Related Impairment questionnaires, most (90% of non-PAP users and 100% of PAP users) found the recall period of 7 days to be “easy” to think about, and generally found the questionnaires to be easy to complete. 80% of individuals among both non-PAP users and PAP users reported that the questions within the PROMIS questionnaires were relevant.

**Table 4:** Cognitive debriefing results

	PROMIS Fatigue-8a	PROMIS Sleep- Related Impairment-8a	ESS
**Non-PAP users (*N*** **= 20)**			
Paraphrase correctly?	Range: 85–100%	Range: 83–100%	-
Clear?	Range: 75–100%	Range: 82–100%	75% (15 of 20)
Recall period easy to think about?	90% (18 of 20)	90% (18 of 20)	-
Easy to complete?	75% (15 of 20)	75% (15 of 20)	-
All questions relevant?	80% (16 of 20)	80% (16 of 20)	80% (16 of 20)
Suggested changes	2 would repeat “in the past 7 days” using a larger font to emphasize the timeframe; 1 would make the font larger for all items		2 suggested clarifying whether the question about sitting and talking to someone was in person or on the phone; 3 suggested clarifying whether sitting quietly after a meal was alone or with others
**PAP users (*N*** **= 10)**			
Paraphrase correctly?	100%	100%	-
Clear?	Range: 60–100%	Range: 60–100%	70% (7 of 10)
Recall period easy to think about?	100% (10 of 10)	100% (10 of 10)	-
Easy to complete?	80% (8 of 10)	80% (8 of 10)	-
All questions relevant?	80% (8 of 10)	80% (8 of 10)	90% (9 of 10)
Suggested changes	1 would remove the column before each item containing coding numbers because they were distracting; 1 would put “in the past 7 days” in bold; 1 suggested asking each question before and after the use of PAP to determine changes		-

The concepts and symptoms identified during the concept elicitation portion of the interview were mapped to the content of the PROMIS Fatigue-8a, PROMIS Sleep-Related Impairment-8a, and ESS. These questionnaires primarily cover fatigue and sleepiness. Given that fatigue was reported by 100% of participants and sleepiness by 75–90%, the measures appeared to have good concept coverage. Other concepts were also mentioned frequently during the interviews, such as difficulty concentrating (85% for non-PAP users, 50% for PAP users), dry mouth (60% of both), interrupted sleep (50% of both), and headaches (50% of both).

## Discussion

Fatigue and sleepiness were among the most common symptoms of OSA reported by both non-PAP and PAP users, with fatigue cited most frequently as the most bothersome symptom. Fatigue and sleepiness have long been reported in OSA [12, 15], and are associated with greater comorbidity and poorer HRQoL [46]. Among individuals with OSA, those with excessive daytime sleepiness have higher rates of depression, gastroesophageal reflux disease, asthma, and angina, and lower scores of mental and physical functioning [13]. A study of veterans evaluated for OSA found that higher levels of fatigue were associated with poorer respiratory-related HRQoL, regardless of OSA severity [46]. Fatigue has also been associated with more depression and anxiety and poorer self-reported mental health in OSA patients [16]. 

Participants in the current study reported being negatively impacted in terms of their ability to engage in daily activities, their physical functioning, social functioning, emotional functioning, and relationships with family and friends. Many participants also reported that OSA symptoms such as fatigue impaired their ability to work for pay and be productive at their job, noting that fatigue made it hard to concentrate and that productivity often waned in the afternoon. Similar to the results found in our study, a variety of previous studies have demonstrated the substantial impacts of OSA on health [7, 47, 48] and work productivity [47, 49, 50]. When treatment is successful in reducing the severity of OSA, measured by the number of respiratory events per hour or other measures, there can be positive impacts on memory and executive functioning [51], improved performance at work [52] as well as reductions in symptom severity and improvements in social interactions and daily and emotional functioning [53]. 

In general, participants found the PROMIS Fatigue-8a, Sleep-Related Impairment-8a, ESS, PGI-S Fatigue, and PGI-C Fatigue to be relevant and clear. While some participants indicated that the wording of some questions or response options were “vague,” all participants were able to provide a response, suggesting that any issues with interpretation or clarity were minor. With PROMIS-Fatigue and Sleep Impairment, the 7-day recall period was acceptable, easy to consider, and likely minimized any recall bias. Our research confirms that there are existing PROs that can be used with confidence in clinical practice to monitor how patients are feeling and functioning or included in clinical trials to evaluate the efficacy of new treatments for OSA.

One of the key findings of this research was that participants considered fatigue and sleepiness as two separate concepts, with fatigue emerging as a distinct and robust symptom observed in OSA. During concept elicitation, participants used “tired” and “sleepy” separately when listing symptoms, and both were two of the most common symptoms mentioned. Others have noted the need to distinguish between daytime sleepiness and fatigue [17, 21], suggesting that they are distinct concepts [18, 54], even though fatigue has received less attention clinically and in research [54]. 

There were numerous strengths associated with the study design. The study sample included both PAP and non-PAP users with a confirmed diagnosis of OSA and a heterogenous sample in terms of most demographic characteristics such as gender, education, and household income. All interviews were conducted via an online video conferencing (Zoom), which allowed the interviewer to interpret body language and probe further if necessary. It also allowed for a seamless transition from the concept elicitation to the cognitive debriefing portion of the interview, when participants were shown a copy of each measure to be debriefed, providing instant feedback while completing it. In addition, saturation, the point at which no new concepts were mentioned, was achieved by the 11th interview in non-PAP users and by the 7th interview in PAP users.

The results should be viewed in light of the study’s limitations. First, certain demographic groups were either under-represented or not represented. For example, both groups included a majority of participants with higher levels of education. As another example, PAP users included only two females and no individuals of Black race or Hispanic ethnicity; only one individual of Asian race was included in either group. However, saturation was achieved, and the primary intent was to explore the PRO measures within non-PAP users (the larger of the two groups). The non-PAP participants most closely reflect those who will be eligible for the planned late phase AD109 clinical trial. The sample size of the PAP group was smaller than the non-PAP group; however, that was by design since the population of primary interest for this study was the non-PAP group. In addition, because participants were recruited from 3 sites in the US, the sample may not have been as geographically diverse as it could have been if sites outside of the US had participated. Future research should focus on evaluating individuals with OSA underrepresented in this study in terms of demographic characteristics and geographic location, to more fully understand the experience of individuals with OSA.

Another limitation of this study was that all participants provided a rating of at least “mild” fatigue on the PGI-S; different results may have been obtained if individuals without fatigue had been included. Additionally, due to the number and length of the questionnaires, not all items could be cognitively debriefed (e.g., individual items from the ESS were not debriefed). Therefore, not all the participants’ opinions regarding the items may be reflected in the results. However, all items in the key measures (PROMIS Fatigue 8a, PROMIS Sleep-Related Impairment − 8a, PGI-S Fatigue, PGI-C Fatigue) were debriefed in full. Finally, due to the length of the interview, not all interview questions could be asked of all participants. It is possible that different results may have been obtained if all questions had been asked.

## Conclusions

All participants reported fatigue, which represents a distinct and often more bothersome concept from sleepiness. Our results reinforce the underlying concept that fatigue is highly prevalent in OSA, is often the most important OSA symptom for those individuals who experience it, and should receive more attention in both research studies and clinical practice. Fatigue and sleepiness emerged as two distinct concepts. Symptoms associated with OSA can significantly hinder daily activities, limit physical functioning and contribute to negative emotional states, resulting in strained relationships and social functioning. In real-world clinical practice, it is paramount to assess fatigue as well as sleepiness when evaluating the detrimental impact of OSA on individuals. The PROMIS Fatigue 8a, PROMIS Sleep-Related Impairment 8a, ESS, PGI-S Fatigue, and PGI-C Fatigue demonstrated that they are clear, and relevant for those with OSA, and are appropriate for use in clinical settings and research.

## Data Availability

The datasets used and/or analyzed during the current study are available from the corresponding author on reasonable request.

## Abbreviations

ESS: Epworth Sleepiness Scale
HRQoL: Health-related quality of life
OSA: Obstructive sleep apnea
PAP: Positive airway pressure
PGI-C: Patient-Global Impression of Change
PGI-S: Patient-Global Impression of Severity
PRO: Patient-reported outcome
PROMIS: PRO Measurement Information System
